# Deregulation of the Glymphatic System in Alzheimer’s Disease: Genetic and Non-Genetic Factors

**DOI:** 10.14336/AD.2023.1229

**Published:** 2023-12-29

**Authors:** Yan-Hong Hu, Ting Su, Lin Wu, Jun-Fang Wu, Dan Liu, Ling-Qiang Zhu, Mei Yuan

**Affiliations:** ^1^Department of Neurology, The Second Affiliated Hospital, Hengyang Medical School, University of South China, Hengyang, Hunan 421001, China.; ^2^Department of Pathophysiology, School of Basic Medicine, Tongji Medical College, Huazhong University of Science and Technology, Wuhan, Hubei 430030, China.

**Keywords:** Alzheimer’s disease;, risk factors, glymphatic system, meningeal lymphatic vessels, blood-brain barrier

## Abstract

Alzheimer’s disease (AD) is the most prevalent form of dementia and is characterized by progressive degeneration of brain function. AD gradually affects the parts of the brain that control thoughts, language, behavior and mental function, severely impacting a person’s ability to carry out daily activities and ultimately leading to death. The accumulation of extracellular amyloid-β peptide (Aβ) and the aggregation of intracellular hyperphosphorylated tau are the two key pathological hallmarks of AD. AD is a complex condition that involves both non-genetic risk factors (35%) and genetic risk factors (58-79%). The glymphatic system plays an essential role in clearing metabolic waste, transporting tissue fluid, and participating in the immune response. Both non-genetic and genetic risk factors affect the glymphatic system to varying degrees. The main purpose of this review is to summarize the underlying mechanisms involved in the deregulation of the glymphatic system during the progression of AD, especially concerning the diverse contributions of non-genetic and genetic risk factors. In the future, new targets and interventions that modulate these interrelated mechanisms will be beneficial for the prevention and treatment of AD.

## 1. Introduction to Alzheimer’s disease

Dementia is a decrease in cognitive abilities across multiple domains significantly impairing independent living, social interaction and occupational performance [1]. Alzheimer’s disease (AD) is the most prevalent form of dementia, characterized by progressive degeneration of brain function, beginning with mild memory loss and potentially leading to an inability to communicate or respond to one’s environment [2]. As the disease progresses, it gradually affects the parts of the brain that control thought, language, behavior, and mental function, severely impacting a person’s ability to carry out daily activities [3]. Ultimately, this can lead to death. AD is rapidly becoming one of the most costly, fatal, and burdensome diseases of this century (Dementia in Europe Yearbook 2019: estimating the prevalence of dementia in Europe. 2020. www.alzheimereurope.org/content/download/195515/1457520/file/FINAL%2005707%20Alzheimer%20Europe%20yearbook%202019.pdf. Alzheimer Europe), currently affecting approximately 50 million people worldwide (World Alzheimer Report 2018. The state of the art of dementia research: new frontiers. www.alzint.org/u/WorldAlzheimerReport2018.pdf. Alzheimer’s Disease International). With the aging of societies and increasing life expectancy, AD is becoming more prevalent. The World Health Organization (WHO) predicts that the number of people living with AD will increase to 82 million by 2030 and could reach 152 million by 2050 (WHO (2017). Dementia. Available online at: www.who.int/news-room/fact-sheets/detail/dementia).

Although the accumulation of extracellular amyloid-β peptide (Aβ) and the aggregation of intracellular hyperphosphorylated tau have been identified as the two key pathological hallmarks of AD [4, 5], its specific pathogenesis and underlying mechanisms are not yet fully understood. AD is a complicated condition that involves both non-genetic risk factors (35%) and genetic risk factors (58-79%) [6]. Non-genetic risk factors are typically associated with sporadic AD (SAD), while genetic risk factors are linked to both familial AD (FAD) and SAD [7-9]. Notably, FAD and SAD differ in terms of the age of onset[10]. AD can also be classified into early-onset AD (EOAD) and late-onset AD (LOAD), which occur before and after the age of 65 and account for 5% and 95%, respectively [8, 9]. Genetic risk factors play a dominant role in the pathogenesis of EOAD but not LOAD [8]. Most cases of AD are caused by a complex interplay of non-genetic and genetic risk factors, in addition to aging [11, 12]. While some risk factors cannot change, such as age and family history, growing evidence shows that modifiable risk factors such as diabetes, hypertension, dyslipidemia, sleep and mental disorders can be closely monitored to prevent cognitive decline and improve quality of life in AD patients [13, 14]. Moreover, studies have suggested that both non-genetic and genetic risk factors can affect the glymphatic system to varying degrees. Thus, researchers are investigating the underlying mechanisms of the deregulation of the glymphatic system during the progression of AD[15], especially the diverse contributions of non-genetic and genetic risk factors. Therefore, identifying novel therapeutic strategies that target the diverse risk factors that cause deregulation of the glymphatic system will be beneficial for clinical research.

## 2. Description of the components of the glymphatic system

Conventional wisdom has long held that the central nervous system (CNS) does not contain lymphatic vessels or lymphatic circulation and lacks the lymphatic pathways to clear brain metabolites. Recent studies have shown that a glymphatic system does exists throughout the entire brain of mice, where it plays an essential role in clearing metabolic waste, transporting tissue fluid, and participating in the immune response [16, 17]. The glymphatic system comprises the glymphatic pathway and meningeal lymphatic vessels (MLVs) [18]. This intricate system carries out the exchange between cerebrospinal fluid (CSF) and interstitial fluid (ISF). Additionally, it is intricately connected to the onset of neurodegenerative diseases, age-related modifications in the brain, traumatic brain injuries, circulatory disorders, and tumors [19].

In 2012, Iliff et al. [16] used two-photon technology to discover the exchange between CSF and ISF and named this the glymphatic pathway. The glymphatic pathway involves the interaction of the endfeet of astrocytes with the walls of blood vessels and operates through the protein aquaporin-4 (AQP4), which is found on the terminal feet of astrocytes [16]. This route primarily consists of the periarterial CSF inflow channel, the perivenous tissue fluid outflow channel, and AQP4 on the astrocytes that connects these two channels. When an arterial pulse occurs, CSF influxes into the brain parenchyma through the periarterial space and exchanges with the ISF via astroglial AQP4 water channels [20, 21]. This exchange facilitates the transportation of various metabolites into the perivenous space and eventually into the circulation of CSF or cervical lymphatic vessels [22]. Besides arterial pulsation, other factors, such as respiration, sleep, head posture, and intracranial pressure, are also believed to influence fluid exchange and flow in this pathway [23, 24]. The glymphatic pathway is crucial for clearing various substances from the CNS, including Aβ [16], tau [25], lactate [26], apolipoprotein E (APOE) [27], and lipids [28]. It plays an important role in the progression of CNS diseases, particularly neurodegenerative diseases [19].

**Figure 1.**
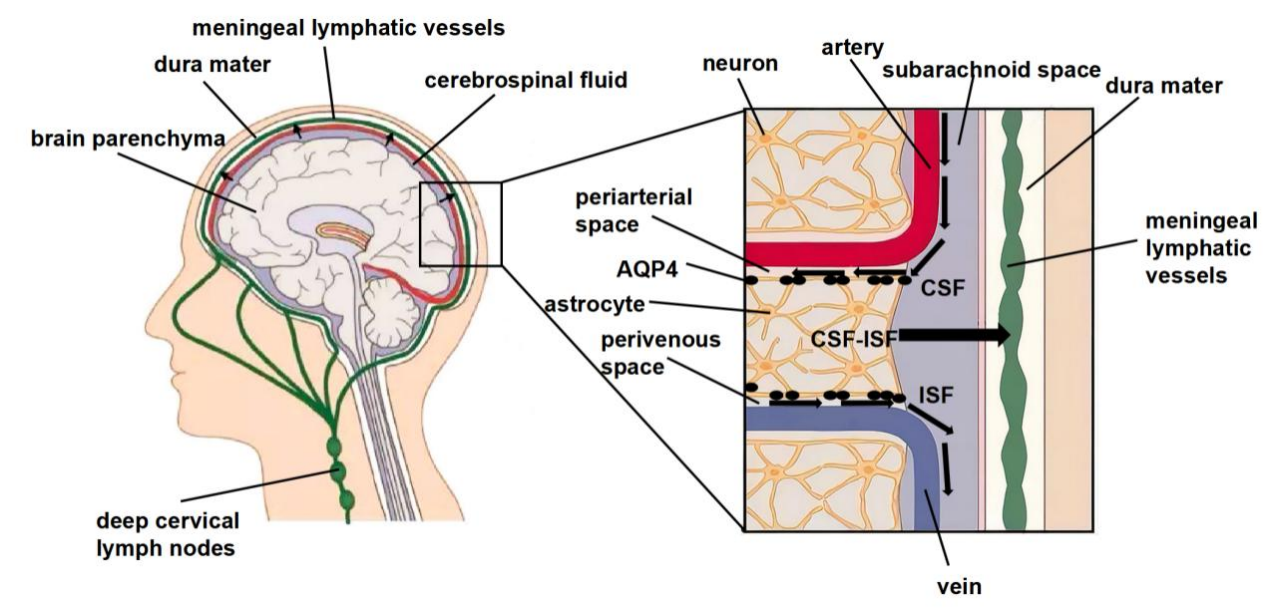
**The glymphatic system**. The glymphatic pathway, meningeal lymphatic vessels (MLVs) and deep cervical lymph nodes (DCLNs) establishes a novel drainage system, named the glymphatic system. Abbreviations: CSF: cerebrospinal fluid; ISF: interstitial fluid; AQP4: aquaporin-4.

Recently, MLVs were found in the dura mater, where they directly drain CSF to deep cervical lymph nodes (DCLNs) [29, 30]. Debate has arisen regarding the underlying mechanisms of this phenomenon. So far, the consensus is that the combined action of the glymphatic pathway, MLVs, and DCLNs establishes a novel drainage system (Fig. 1), which plays a pivotal role in facilitating CSF circulation and the efficient removal of cerebral metabolic waste [18]. In 2017, Absinta et al. [31] utilized magnetic resonance imaging (MRI) to observe the presence of MLVs in both humans and primates. This groundbreaking discovery has sparked global interest, leading to numerous studies exploring the potential connection between MLVs and CNS diseases. A study has shown that MLVs develop and form after birth [32]. Related studies on adult mice have focused on the characteristics of vascular endothelial growth factor receptor-C (VEGFR-C), which is expressed mainly on vascular smooth muscle cells, and vascular endothelial growth factor receptor-3 (VEGFR-3), a receptor tyrosine kinase of VEGF that is expressed mainly in lymphatic endothelial cells. They found that the loss of VEGFR-C or VEGFR-3 can cause regression of MLVs, leading to impaired CSF drainage function, and that excess VEGFR-C can induce the formation of MLVs [32]. These findings indicate that MLVs possess plasticity and regeneration ability that may help them regulate the pathophysiological processes of certain CNS diseases.

## 3. Dysfunction of the glymphatic system in AD

**Figure 2.**
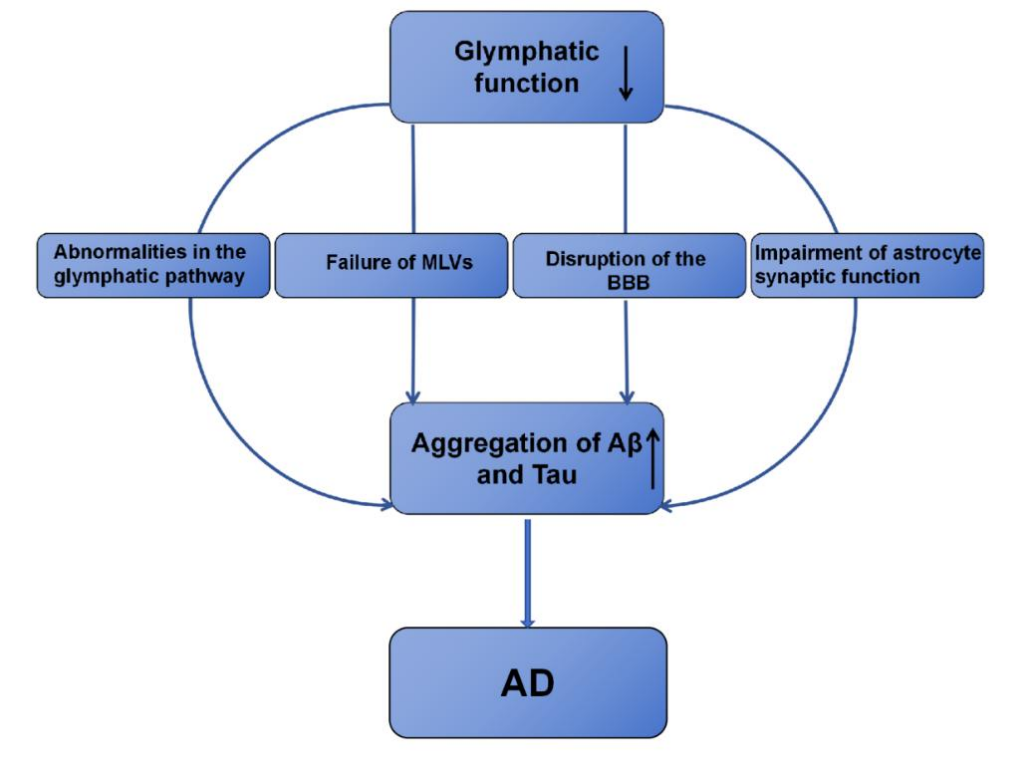
**Dysfunction of the glymphatic system in the development of AD**. Dysfunction of the glymphatic system through various mechanisms, including abnormalities in the glymphatic pathway, failure of MLVs, disruption of the BBB and impairment of astrocyte synaptic function, hinders the clearance of proteins, leading to the accumulation of metabolites in neurons and ultimately contributing to the onset of AD. Abbreviations: MLVs: meningeal lymphatic vessels; BBB: blood-brain barrier; Aβ: amyloid-β peptide; AD: Alzheimer’s disease.

The main pathological changes observed in AD include the accumulation of Aβ to form senile plaques (SPs), the formation of intracellular neurofibrillary tangles (NFTs) caused by the abnormal deposition of excessive tau phosphorylation, and gliosis with neuronal loss [33], while impairment of the protein clearance pathway contributes to abnormal protein deposition. Recent studies have highlighted the importance of the glymphatic system in clearing macromolecular proteins, including Aβ and tau, as well as antigens [16, 25, 26]. Dysfunction of the glymphatic system hinders the clearance of proteins, leading to the accumulation of metabolites in neurons and ultimately contributing to the onset of AD. Dysfunction of the glymphatic system accelerates the development of AD through various mechanisms, including abnormalities in the glymphatic pathway, failure of MLVs, disruption of the blood-brain barrier (BBB) and impairment of astrocyte synaptic function (Fig. 2).

A key protein in the glymphatic pathway is AQP4, which is found in the cell membrane of astrocytic endfeet and serves as the lateral component of the perivascular space (PVS) [16, 25]. Using two-photon microscopy, Rosu et al. [34] discovered that Aβ enters the CSF via AQP4 on astrocytes and travels to peripheral lymphoid tissues. AQP4 plays a crucial role in promoting the glymphatic pathway, and its deletion can reduce the transport and clearance capacity of the glymphatic system [35, 36]. In AQP4-knockout mice, soluble Aβ in the brain increases by 25% to 50% [35]. This increase is not due to changes in the synthesis or degradation of Aβ but rather to a reduction in the clearance of Aβ from the brain caused by lesser inflow of CSF and outflow of ISF. The ability of the brain extracellular space (ECS) to drain solutes is weakened in AQP4-deficient mice, which suggests that the transport of solutes between the PVS and the brain parenchyma is regulated by glial cells, specifically AQP4 [16]. The migration ability of astrocytes in AQP4-knockout AD mice is impaired, and glial cells are not effectively recruited to plaques [37]. From the perspective of cell migration, it suggests that the loss of AQP4 slows the migration ability of astrocyte cell bodies, which hinders the formation of glial networks with microglia around the plaque, and this isolation of the plaque from the surrounding tissues accelerates the aggregation of Aβ and the development of disease pathology [37]. Moreover, there is a phenomenon known as AQP4 depolarization in AD, which is characterized by the displacement of AQP4 on cells, resulting in a decrease in AQP4 on the endfeet membrane and an increase in AQP4 in areas outside the endfeet membrane [38]. This shift disrupts the exchange of CSF and ISF, leading to abnormal clearance of Aβ and phosphorylated tau protein in the brain. The PVS, also referred to as the Virchow-Robin space (VRS), includes the periarterial and perivenous spaces, which are also critical components of the glymphatic pathway, as they provide a low-resistance pathway for CSF-to-ISF flow and play a vital role in clearing waste products from the brain [39, 40]. Dysfunction of paravascular clearance can limit the effective clearance of waste products in the brain, including Aβ and tau, leading to the accumulation of macromolecular proteins and further accelerating the dysfunction of paravascular clearance in a vicious cycle [41]. An enlarged perivascular space (EPVS) can cause glymphatic dysfunction in the brain, which is associated with cerebrovascular disease, cognitive impairment, and other neurological diseases [42]. Multiple studies have utilized the diffusion tensor image analysis along the perivascular space (DTI-ALPS) to assess the movement of water molecules in the direction of the PVS, which reflects the functional activity of the glymphatic system [43, 44].

The discovery of MLVs has offered fresh insights into the pathophysiology of age-related neuro-degenerative conditions. This is because intracranial CSF outflow from MLVs is notably reduced in older mice [45]. This malfunction of MLVs can result in the accumulation of abnormal pathological proteins such as Aβ, tau, and α-synuclein, which can accelerate the progression of neurodegenerative diseases [46]. Moreover, MLVs at the skull base play a crucial role in the uptake and outflow of CSF and are involved in lymphedema [47]. In a recent study, improving meningeal lymphatic drainage combined with anti-Aβ immunotherapy resulted in a significant improvement in both pathological and cognitive symptoms in mice [48]. These findings emphasize the potential of regulating MLVs as a therapeutic strategy for neurological diseases caused by the buildup of intracranial macromolecular metabolic waste.

The BBB and glymphatic system have similar clearance mechanisms, with approximately 25% of Aβ cleared through the BBB [49]. BBB injury affects glymphatic function through basement membrane thickening and narrowing of the PVS, which in turn reduces the flow of CSF and ISF, ultimately resulting in a decrease in the effective transport rate of the PVS [50]. However, glymphatic dysfunction also affects the BBB, leading to protein deposition and the progression of AD and cerebral small vessel disease[51]. The glymphatic system and BBB are interconnected in a harmful cycle, the disruption of one pathway leading to negative consequences for the other [52].

Astrocytes play a crucial role in supporting and guiding neurons, as well as promoting synaptic connections and ensuring the survival of neurons [53]. They are located between the cell bodies of nerve cells and their protuberances, where they regulate neural circuit function in the developing and adult brain [53]. During brain development, astrocytes contribute to circuit development through diverse mechanisms, including the induction, elimination, and maturation of neuronal synapses. In the adult brain, astrocytes are essential for maintaining the neuronal environment and are involved in processes such as recycling neurotransmitters from the synaptic cleft, maintaining the integrity of the BBB, and regulating energy homeostasis. Additionally, astrocytes play a role in the fluid clearance pathway of the glymphatic system, which is responsible for removing approximately 65% of Aβ from the brain in AD [54]. These findings suggest that astrocyte dysfunction may contribute to the development or progression of various neurodegenerative diseases, possibly by impairing the glymphatic system and synaptic function.

## 4. Role of non-genetic risk factors in glymphatic system disorders in AD

### 4.1 Aging

Numerous factors contribute to the increased risk of AD. Aging is the most significant risk factor for LOAD, which accounts for more than 95% of all AD cases[55]. Likewise, the function of the glymphatic system declines significantly with age [56], which decline is mainly associated with AQP4 depolarization and malfunction of MLVs [41, 45]. Kress et al. [41] demonstrated that there was a significant decrease in CSF tracer (Dex-3 and OA-45) flow into brain tissue in old mice (18-20 months old) compared to young mice (2-3 months old). The clearance of exogenous Aβ is reduced by 40% in aged mice. Additionally, researchers have observed a decrease in the polar distribution of AQP4 in the PVS of aged mouse brains [41]. Studies on aging models in both animals and patients have revealed a negative correlation between changes in AQP4 expression and a decrease in glymphatic system function [57, 58]. With age, the choroid plexus produces less CSF, the arachnoid villi decline in number, and arterial wall sclerosis weakens the pulse. These factors collectively contribute to impairments in glymphatic system function during aging [41, 47]. This dysfunction is an important feature of brain aging and can result in a decrease in brain parenchymal Aβ clearance, leading to aging-related neurodegenerative diseases. Moreover, research has indicated that aging mammals might exhibit MLVs dysfunction and decreased CSF drainage capability [45, 47]. These factors can accelerate the accumulation of toxic Aβ proteins in the brain parenchyma, further aggravating AD-related pathology [59]. Peng et al. [60] reported that glymphatic transport is suppressed in AD mice and is more prominent in aged AD mice. Earlier research highlighted a noteworthy decrease in meningeal lymphatic drainage in aged mice (20-24 months old) compared to young mice (2 months old) [46]. Older mice exhibit only half the drainage capacity of younger mice [46]. This diminished drainage efficiency is possibly linked to age-related lymphedema of MLVs, impairing CSF drainage [47]. Moreover, the cerebrovascular basement membranes in the aging brain undergo several changes, such as thickening, reduplication, and vacuolization, which can result in arterial stiffness and reduced arterial pulsatility in the cerebral cortex of aged mice. As a consequence, the function of the glymphatic system is also diminished, potentially leading to the accumulation and deposition of toxic solutes, including Aβ [61, 62].

Therefore, aging could lead to AQP4 depolarization and malfunction of MLVs, ultimately contributing to the deregulation of the glymphatic system and the onset of AD. While reversing the process of aging is not possible, future research will focus on identifying ways to diagnose and intervene in diseases at an early stage by assessing the function of the glymphatic system in aging individuals.

### 4.2 Sleep disorders

Disruption of regular sleep patterns is a common risk factor linked to neurodegenerative diseases such as AD and Parkinson’s disease. An irregular sleep-wake cycle is thought to contribute to the accumulation of waste products in the CNS [63]. Xie et al. [64] proposed that the glymphatic system most actively eliminates brain waste proteins during sleep, particularly during non-rapid eye movement (NREM) sleep and high slow-wave activity. By increasing the quality of sleep and enhancing the glymphatic system’s clearance of abnormal proteins from the brain, we might decrease the likelihood of neurodegenerative diseases [15]. The sleep-enhancing glymphatic system operates through three primary mechanisms. First, during sleep, the body’s adrenergic activity falls, resulting in an expansion of the extracellular space in the brain and a reduction in CSF flow resistance within the brain parenchyma [64]. This phenomenon improves the clearance efficiency of the glymphatic system. Second, the volume of CSF increases during sleep, which enhances the driving force involved in clearance [65]. Finally, the polarization of AQP4 on the paravascular terminal feet of astrocytes is heightened during sleep, facilitating the flow of CSF from the arterial PVS into the brain parenchyma, thereby assisting in the removal of metabolic waste [66]. Consequently, sleep may become a crucial modifiable factor capable of influencing the progression of neurodegenerative diseases. Sleep disorders include sleep deprivation (SD), reduced NREM sleep and obstructive sleep apnea-hypopnea syndrome (OSAHS), all of which can cause glymphatic system dysfunction and promote AD progression through different mechanisms.

SD, defined as too little sleep, can arise from various factors. Numerous studies have extensively explored the impact of SD on protein accumulation within the CNS, which contributes to the onset of AD [67]. Lucey et al. [68] reported increased Aβ levels in the CSF of individuals with SD. Research utilizing positron emission tomography to measure CSF proteins has shown elevated levels of Aβ in CSF, along with increases in tau and α-synuclein in the right hippocampus and thalamus, in individuals following just one night of acute SD [67, 69]. Additionally, disruptions in slow-wave sleep have also been linked to elevated CSF Aβ levels [70]. SD has also been found to cause abnormal synaptic activity in astrocytes of the glymphatic system, leading to the release of braided tau protein from cells and inhibiting its clearance, resulting in the spread of the protein in the brain [67]. This leads to progressive synaptic and neuronal dysfunction, ultimately promoting the progression of AD. Liu et al. [71] used two-photon in vivo imaging to investigate the impact of chronic SD on the glymphatic system in mice. Their findings indicated reduced solute clearance efficiency in the PVS and loss of AQP4 polarity in the cerebral cortex. Eide et al. [72] conducted experiments on human SD and discovered that complete SD led to an increase in the content of tracers in CSF in the brain. Their findings provide the first evidence that SD impairs molecular clearance in the human brain. However, partial SD with preserved slow-wave sleep did not affect Aβ levels, reinforcing the critical role of slow-wave sleep in facilitating metabolite clearance [73]. Consequently, SD can potentially exacerbate AD progression by compromising astrocyte synaptic function, depolarizing AQP4, and expanding the PVS, thereby affecting the glymphatic pathway and undermining its function.

The clearance by the glymphatic system is periodically regulated by the sleep-wake cycle, its function being activated during sleep [64]. Electroencephalogram (EEG) monitoring has demonstrated that the glymphatic system is most efficient during slow-wave activity in NREM sleep [74]. NREM sleep is characterized by deep sleep stages dominated by slow waves. Interestingly, during these stages, glial cells in the brain shrink in volume by approximately 60%, creating more room for CSF to clear metabolic waste [75]. The rhythmic pulsations of deep NREM sleep foster the elimination of waste at rates 10 to 20 times greater than those during wakefulness [75]. Reduced NREM sleep is associated with a reduction in the volume of the cerebral cortex and subcortical regions coupled with an increase in the volume of white matter hyperintensity lesions [76]. And tau protein increases in the brain and CSF, as does the level of Aβ in the CSF [77]. Hence, even if sleep duration is sufficient, poor sleep quality leading to reduced deep sleep could result in heightened levels of tau and Aβ proteins in the brain, contributing to the onset and progression of AD.

OSAHS is often associated with sleep fragmentation and alterations in sleep structure [78]. If left untreated, OSAHS patients may have Aβ accumulation over time, which can ultimately lead to a greater risk of developing dementia and AD [78]. Moreover, OSAHS may negatively impact the clearance of Aβ through the glymphatic system. Lee et al. [79] utilized DTI-ALPS to assess the function of the glymphatic system in both OSAHS patients and healthy controls and found that OSAHS patients exhibited dysfunction in their glymphatic system, which was closely correlated with the severity of their condition and the oxygen saturation index during NREM sleep. OSAHS may lead to glymphatic dysfunction through changes in sleep patterns and multiple episodes of hypoxia[80]. The acute hypoxic phase reduces water diffusion parallel and perpendicular to the fibers, resulting in a decreased diffusion coefficient in the PVS [81]. The respiratory movement of OSAHS patients during apnea can lead to increased intrathoracic and intracranial pressure, as well as sudden drops in pressure at the end of apnea. These repeated high-pressure fluctuations may hinder lymphatic flow, resulting in impairment of glymphatic system function[82]. Moreover, OSAHS may contribute to systemic inflammation through various pathways, including dysfunction of the BBB, elevated cytokine levels, and oxidative stress resulting from impaired convective flow and CSF-to-ISF conversion [82]. These factors can impede waste removal processes, leading to further complications.

In conclusion, sleep disorders, such as SD, reduced NREM sleep and OSAHS, can cause synaptic impairment in astrocytes, depolarizing AQP4 and expanding the PVS, ultimately contributing to the deregulation of the glymphatic system and the onset of AD. Future research should investigate whether the mitigation of sleep disorders plays a crucial role in maintaining effective glymphatic clearance and mitigating the occurrence and progression of AD.

### 4.3 Metabolic diseases

Metabolic diseases such as hypertension, diabetes, and dyslipidemia are known risk factors for AD, specifically SAD. These factors can impact the metabolism and accumulation of Aβ and tau proteins in the brain through various mechanisms, ultimately resulting in neuronal dysfunction and death, thereby triggering the development of AD.

Hypertension affects the normal fluid dynamics of CSF by inhibiting the movement of vascular pumps and the substance clearance function of the glymphatic system, which can lead to the development of AD [83]. The flow of CSF in the brain is synchronized with the heartbeat and depends strongly on the curvature of the arterial walls. When mice are induced to develop high blood pressure, this flow became less efficient and slowed[84]. Pulsation plays a crucial role in regulating the glymphatic system by facilitating the transportation of fluid and solutes in the PVS [62]. In hypertensive patients, the pulse rate and cerebral perfusion may be weakened, potentially resulting in impaired glymphatic system clearance and contributing to the accumulation of Aβ around blood vessels, which leads to the onset of AD [85].

Diabetic patients often suffer cognitive dysfunction [86]. A study conducted on a drug-induced middle-aged rat diabetes model revealed that diabetes can worsen hippocampus-related cognitive decline [87]. Further research has demonstrated that in this model, the clearance capacity of the glymphatic system in the hypothalamus and hippocampus is significantly reduced, which is highly correlated with learning and memory impairment [87]. Astrocyte activation in the hippocampus, loss of AQP4 in the perivascular area, and microcirculation disturbance are believed to be the underlying causes [87, 88]. MRI analysis of diabetic rats revealed that the clearance of gadopentetate meglumine, a contrast agent, from the interstitial space in the hippocampus was 3 times slower in diabetic rats than in nondiabetic rats [88]. This was confirmed by fluorescence imaging analysis, which also showed a strong negative correlation between cognitive defects and the retention of contrast agents and fluorescent tracers [88]. These findings suggested that the glymphatic system plays a crucial role in diabetes-related cognitive decline and is expected to become a therapeutic target.

Dyslipidemia can increase the risk of developing AD through the formation of atherosclerotic plaques in cerebral blood vessels caused by hyperlipidemia, which can negatively impact brain function. Plaques can lead to arterial wall stiffness and reduced pulsatility, ultimately affecting cerebral hemodynamics. Importantly, arterial pulsation plays a crucial role in driving the glymphatic system [62], which may be one of the causes of dyslipidemia-triggered AD.

Hence, metabolic diseases, such as hypertension, diabetes, and dyslipidemia, can cause AQP4 depolarization, further resulting in dysfunction of the glymphatic system and triggering the development of AD. One potential direction for large-scale clinical research is to focus on changing living habits and reducing the occurrence of metabolic diseases, which can enhance the glymphatic clearance of waste and potentially reduce the prevalence of neurodegenerative diseases such as AD.

### 4.4 Cerebrovascular diseases

Cerebrovascular diseases can be classified into two main types according to their pathology: hemorrhagic and ischemic cerebrovascular diseases, which can directly lead to AD. Hemorrhagic cerebrovascular diseases include subarachnoid hemorrhage and cerebral hemorrhage, while ischemic cerebrovascular diseases include cerebral infarction and cerebral small vessel disease. The glymphatic system is impaired in cerebrovascular diseases.

In subarachnoid hemorrhage model mice, blood components such as fibrinogen are present in the EPVS, the distribution of AQP4 around blood vessels is reduced, and a neuroinflammatory response is obvious [89, 90]. Further evaluation of the glymphatic system in a subarachnoid hemorrhage model revealed that the mice had less clearance of the contrast agent gadolinium than the normal control group after injection of the contrast agent for 1 hour [90]. Another study showed an increase in both total tau protein and phosphorylated tau protein after 7 days of subarachnoid hemorrhage, indicating a decrease in the clearance of glymphatic system proteins [89]. On the other hand, after subarachnoid hemorrhage occurs, blood clots may obstruct MLVs and impair drainage function [46]. Consequently, there may be excessive accumulation of toxic and inflammatory metabolites, which could lead to long-term brain damage. These findings suggest that following subarachnoid hemorrhage, there is blockage in the PVS, abnormalities in the polarity distribution of AQP4, and dysfunction in the drainage of MLVs. This leads to a decrease in the ability of the glymphatic system to clear abnormal proteins, ultimately resulting in cognitive impairment after cerebral hemorrhage.

In a mouse model of ischemic stroke, pathological changes similar to those observed in subarachnoid hemorrhage, including PVS expansion, loss of polarity distribution of AQP4, and astrocyte disorders surrounding ischemic lesions, can be observed [91]. These changes indicate an abnormality in the structure of the glymphatic system. The function of the glymphatic system evaluated in animal models reveals a decrease in both inflow and clearance functions in the short term after stroke [57]. Furthermore, the function of this system can be restored via spontaneous arterial recanalization after embolic ischemic stroke [92]. Dysfunction of the glymphatic system may explain the abnormal protein deposition in the brain after ischemic stroke and the development of dementia. However, the molecular mechanism remains to be confirmed by further research.

Cerebral amyloid angiopathy (CAA) is a common cerebral small vessel disease characterized by the deposition of Aβ on the leptomeninges and walls of small vessels, leading to cognitive dysfunction in elderly individuals [93]. A study of rats with CAA revealed that the normal fluid transport of the glymphatic system was disrupted. This impairment may be due to the ineffective clearance of brain metabolites, such as Aβ, leading to their accumulation in blood vessel walls and the brain, ultimately resulting in a decline in cognitive function[94]. As CAA progresses, Aβ clearance decreases, and deposition becomes more prominent with age, exacerbating the pathological changes of CAA and further impairing the function of the glymphatic system [95]. These findings highlight the vicious cycle through which CAA aggravates glymphatic system dysfunction, and timely intervention in the glymphatic system could be a potential therapeutic approach for CAA and CAA-related cognitive decline.

Therefore, cerebrovascular diseases, such as subarachnoid hemorrhage and ischemic stroke, can cause AQP4 depolarization, expansion of the PVS, dysfunction in the drainage of MLVs and synaptic impairment, further disturbing the function of the glymphatic system and facilitating the progression of AD. Further research on the molecular mechanisms underlying the relationships between cerebrovascular diseases and the glymphatic system is urgently needed.

### 4.5 Traumatic brain injury

Traumatic brain injury (TBI) has been identified as an independent risk factor for neurodegeneration, including dementia and AD [96, 97]. Glymphatic system dysfunction plays an important role in pathological processes following TBI [25]. In mice, TBI results in an increase in the overall expression of AQP4 in the cortex and striatum but a decrease in its distribution around blood vessels [25]. Additionally, the polar distribution of AQP4 does not recover in most mice even after 4 weeks. Simultaneously, the impairment of the transport and clearance of tau protein in the glymphatic system persists for up to 4 weeks after injury, leading to a general increase in phosphorylated tau protein in the brain. Researchers performed the same experiment in AQP4-knockout mice and showed that a lack of AQP4 worsened the accumulation of phosphorylated tau in the brain after TBI [25]. In a study on a mouse model of mild TBI, Bolte et al. [98] discovered that TBI can hinder the drainage of MLVs in normal mice, which can exacerbate central inflammation and cognitive impairment. The cause of this hindrance is believed to be TBI-induced brain tissue edema and increased intracranial pressure, which compresses the MLVs [98]. These findings reveal that a malfunctioning glymphatic system can lead to reduced protein clearance and abnormal protein aggregation, which can contribute to the secondary degeneration observed after TBI.

TBI causes AQP4 depolarization and malfunction of MLVs, further resulting in dysfunction of the glymphatic system and triggering the development of AD. Thus, restoring the drainage function of the glymphatic system is crucial for continuously regulating the brain microenvironment and improving long-term prognosis following TBI. It also represents a key area of research for future studies.

### 4.6 Psychological disorders

Depression is one of the most prevalent mood disorders and is often accompanied by cognitive decline [99]. Observations of glymphatic function in a chronic unpredictable mild stress (CUMS) mouse model revealed that exposure to CUMS led to depression-like and amnesic symptoms. This change was coupled with glymphatic dysfunction, reduced concentrations of neurotransmitters, and upregulation of markers of neuroinflammation in the nervous system [100]. Furthermore, CUMS-exposed mice exhibited attenuated middle cerebral artery pulsatility, decreased vascular compliance, and depolarized expression of AQP4, confirming dysfunction within the glymphatic system [100]. These findings suggest a potential connection between depression and cognitive decline through the disruption of glymphatic system function. Researchers have successfully rescued potential glymphatic system destruction and protected cerebrovascular function by supplementation with polyunsaturated fatty acids (PUFAs), which have shown promising results in rescuing depression and related cognitive dysfunction [100].

Consequently, psychological disorders, mainly depression, cause depolarized expression of AQP4, further leading to dysfunction of the glymphatic system and contributing to the development of AD. Notably, few treatment options specifically address the cognitive deficits associated with depression, and even fewer have demonstrated clinical efficacy. Therefore, gaining a deeper understanding of the connection between the glymphatic system and depression will be crucial in the development of novel and more effective therapeutic approaches to treat depression and its accompanying cognitive decline.

## 5. Role of genetic risk factors in glymphatic system disorders in AD

In addition to non-genetic risk factors, genetic risk factors are implicated in glymphatic system dysfunction in AD. Heredity is a significant factor in the occurrence of AD. Two types of genetic variants have been associated with AD: pathogenic variants and risk variants. Pathogenic variants are gene mutations that directly lead to the occurrence of AD, particularly familial EOAD. The three known pathogenic genes include the amyloid precursor protein (APP) gene on chromosome 21, the presenilin 1 (PS1) gene located on chromosome 14, and the presenilin 2 (PS2) gene located on chromosome 1 [101]. Mutations in these genes affect the processing and clearance of Aβ, resulting in abnormal deposition of Aβ in the brain and the formation of plaques [102]. On the other hand, risk variants are specific genetic polymorphisms that either increase or decrease the probability of developing AD, mainly in sporadic LOAD patients. One of the most well-known risk variant genes is APOE. Other genes may also be associated with AD, such as clusterin (CLU), complement receptor-1 (CR1), bridging integrator 1 (BIN1), phosphatidylinositol binding clathrin assembly protein (PICALM), sortilin-related receptor L (SORL1), and triggering receptor expressed on myeloid cells 2 (TREM2) [103]. These genes might contribute to the pathogenesis of AD by affecting dopamine and potassium channels, the transport of Aβ and tau, the immune response, and lipid metabolism pathways [104]. The human APOE gene encodes three isoforms: APOE2, APOE3, and APOE4. APOE2 comprises approximately 10% to 15% of the APOE alleles in the human population, APOE3 70% to 80%, and APOE4 5% to 10%[105]. APOE4 is the most significant genetic risk factor for AD, APOE3 is a neutral genetic risk factor, and APOE2 is a protective factor [105]. Glial cells secrete APOE, which stimulates neurons to produce Aβ [106]. APOE4 results in the highest Aβ production, followed by APOE3 and APOE2 [106]. The APOE2 protein has a strong affinity for Aβ, facilitating its transportation, while the interaction between APOE4 and Aβ is weaker, leading to the accumulation of Aβ and promoting the formation of senile plaques [106]. Thus, APOE4 allele carriers are the population at the highest risk for sporadic AD [107]. Both pathogenic variant and risk variant genes have potential associations with the glymphatic system. Therefore, further research on early intervention targeting the enhancement of glymphatic system function in individuals carrying these high-risk genes could help prevent the onset of AD.

### 5.1 APP, PS1 and PS2

The disease-causing genes APP, PS1, and PS2 directly contribute to the development of AD by accelerating the accumulation of the Aβ protein. In mouse models with these genes, AQP4 polarization disruption has been noted during pathological progression, exacerbating the reduction in synaptic protein expression [35]. This synaptic impairment in the hippocampus and cerebral cortex underlies cognitive decline in AD [108]. In a recent study, researchers evaluated the effectiveness of the glymphatic system in a transgenic Tet-Off APP mouse model of amyloidosis [109]. They observed a disruption of glymphatic function, specifically a decrease in brain-fluid influx, in the Tet-Off APP mouse model. The researchers hypothesize that this hindered circulation of CSF may be due to widespread astrogliosis and amyloid-related blockage of the usual glymphatic flow pathway, leading to redirection toward caudal regions.

Consequently, patients harboring APP, PS1, or PS2 genes are more likely to exhibit AQP4 depolarization, further impairing the function of the glymphatic system and promoting the occurrence of AD. In the future, early intervention and treatment should be implemented for these patients by regulating the function of the glymphatic system.

### 5.2 APOE4 and BIN1

The role of astrocyte-derived APOE4 in the pathogenesis of AD has been extensively studied. APOE4 can contribute to the occurrence and progression of AD by promoting the deposition of Aβ. Bell et al. [110] found that the expression of APOE4, but not APOE2 or APOE3 or deficiency of APOE4, results in breakdown of the BBB and further facilitated the accumulation of Aβ in mice. This breakdown is mediated by the proinflammatory factor CypA-nuclear factor-kB-matrix-metallo-proteinase-9 (CypA-MMP9), which is localized in vascular pericytes. Zlokovic’s team [111] discovered that APOE4 has a detrimental effect on the BBB, allowing harmful substances to enter the brain’s memory and cognitive function areas, which is independent of Aβ and tau proteins and has direct impacts on cognition. Further analysis revealed increased levels of soluble platelet-derived growth factor receptor-β (sPDGFRβ) in the CSF of APOE4 carriers, which was associated with increased BBB permeability in specific brain regions, such as the hippocampus and parahippocampal gyrus. Researchers have shown that the proinflammatory CypA-MMP9 pathway is involved in this process and believe that APOE4 activates the CypA-MMP9 pathway, leading to breakdown of the BBB and impaired neuronal and synaptic function. Blocking the CypA-MMP9 pathway in APOE4 mice restores the integrity of the BBB [110]. Therefore, the use of CypA inhibitors may improve BBB integrity and reduce neuronal and synaptic defects in APOE4 carriers, ultimately slowing the progression of cognitive impairment. Damage to the BBB can further contribute to malfunction of the glymphatic system, potentially explaining the development of AD resulting from APOE4-induced damage to the BBB. Further investigations are needed to determine the molecular mechanism underlying this process. Furthermore, mice harboring APOE4 exhibit lymphatic vessel sclerosis, which impairs the contractile function of MLVs and reduces lymphatic flow [112]. This blockage hinders the normal flow of ISF and CSF, ultimately leading to impaired glymphatic system function.

The BIN1 gene has also been identified as a significant risk locus for AD [113]. Specifically, the BIN1 rs744373 polymorphism has been found to affect tau clearance, potentially explaining the neural mechanism that links BIN1 to the risk of developing AD [114]. This genetic variant presents two possible alleles, A (major allele) and G (minor allele), the G allele being associated with AD and considered the risk allele [115]. Studies have explored the relationship between EPVS and the BIN1 rs744373 polymorphism, finding that individuals carrying the G allele have a greater risk of EPVS than those carrying the A allele [116]. BIN1 may contribute to the progression of AD by initiating EPVS, which in turn leads to disorders of the glymphatic system.

In conclusion, APOE4 results in the breakdown of the BBB and malfunction of MLVs, and BIN1 causes the expansion of PVS, further leading to the accumulation of Aβ and tau proteins and the proliferation of microglia, thereby accelerating the progression of AD. Further investigation into the correlation between AD-related genes and the glymphatic system is imperative. Regulating the function of the glymphatic system holds great potential for mitigating the risk for individuals carrying these genes.

## 6. Clinical research status of the glymphatic system in cognitive decline patients

Early studies on the glymphatic system mainly concentrated on animal research. To extend these findings to humans, the main approach used to evaluate the function of the glymphatic system in humans is dynamic contrast-enhanced MRI (DCE-MRI) [117]. This technique involved the intrathecal injection of an MRI contrast agent, which served as a CSF tracer, and the change in T1-weighted signals over time was measured to determine the time needed for tracer clearance. This approach enabled the assessment of glymphatic function in different brain regions. MRI of the glymphatic system reveals a slower clearance of contrast agent in human patients with normal intracranial pressure hydrocephalus than in normal controls [118], and this slower clearance is closely associated with the extent of cognitive impairment. Additionally, clearance by the glymphatic pathway and MLVs is impaired in the aging human brain [119]. Similarly, positron emission tomography (PET) has also been utilized to investigate CSF transport into the brain parenchyma as well as efflux pathways [120]. PET has also demonstrated a decrease in CSF clearance of Aβ and tau protein tracers in patients with AD compared to healthy controls [121]. However, these methods involve the invasive injection of contrast agents or radionuclides. In recent years, researchers have increasingly utilized the noninvasive DTI-ALPS method to investigate glymphatic system function. The DTI-ALPS method does not require contrast enhancement and can evaluate the hydromechanics of the glymphatic system by quantifying the diffusion rate of water molecules in different directions. Taoka et al. [122] first proposed the DTI-ALPS method and concluded that the ALPS index reflects the function of the glymphatic system, a decrease in the index indicating an impaired diffusion rate of the PVS and thereby impaired glymphatic system function. Several clinical studies have consistently demonstrated the reliability of the ALPS index as a method for evaluating glymphatic function. The ALPS plays a crucial role in the cognitive dysfunction associated with the deposition of Aβ and tau proteins, suggesting a possible connection between glymphatic system dysfunction and the pathogenesis of AD [43]. In individuals with cerebral small vessel disease, the ALPS index has been independently linearly correlated with overall cognitive function, executive function, attention function, and memory [123]. Other clinical studies have reported a decreased ALPS index in patients with ischemic stroke [124], OSAHS [79], or sleep disorders [125]. The ALPS index serves as a quantitative metric for assessing glymphatic system function and is considered an emerging neuroimaging biomarker. Future clinical studies should recruit large samples from multiple centers to strengthen the evidence on the ALPS index and explore its predictive role in determining the efficacy of various treatment options and its influence on disease prognostic factors. It is also important to identify simpler blood markers that can assess glymphatic function in neurological diseases, which will be helpful in the early identification of clearance impairment and early intervention in this disease.

## 7. Strengths, limitations, and major challenges

The cause of AD is complex, and the specific mechanism of this disease has not yet been determined. Consequently, effective treatments are lacking. This review explored the impact of glymphatic system dysfunction on the progression of AD and explored the potential novel mechanisms through which both genetic and non-genetic risk factors contribute to glymphatic system dysfunction. This article offers a fresh perspective on the relationship between AD and the glymphatic system, shows the great importance of the glymphatic system in AD, and highlights the significant scientific and clinical implications of the glymphatic system in the diagnosis and treatment of AD. Notably, current research on the glymphatic system is primarily based on animal experiments and lacks large-sample trial data. The investigation of its role and mechanism in CNS diseases is still in the early stages, so a comprehensive understanding of this topic awaits further research. Currently, drugs are the primary clinical method for treating AD. Their main focus is to preserve the function of degenerated neurons, and they have been effective at treating patients with early-stage AD. However, the subtle onset of the disease makes it difficult to detect, resulting in most patients initiating treatment during the middle to late stages of the disease. Consequently, the current drugs have suboptimal effects, as they can only alleviate symptoms or slow the progression of the disease without reversing cognitive dysfunction. Given the significant increase in the number of AD patients and the recent failure to develop new drugs, there is an urgent need to identify new treatment targets. Understanding the underlying mechanisms of risk factors is crucial for revealing the development of AD and can offer fresh insights for the diagnosis, treatment, and prevention of this disease.

**Figure 3.**
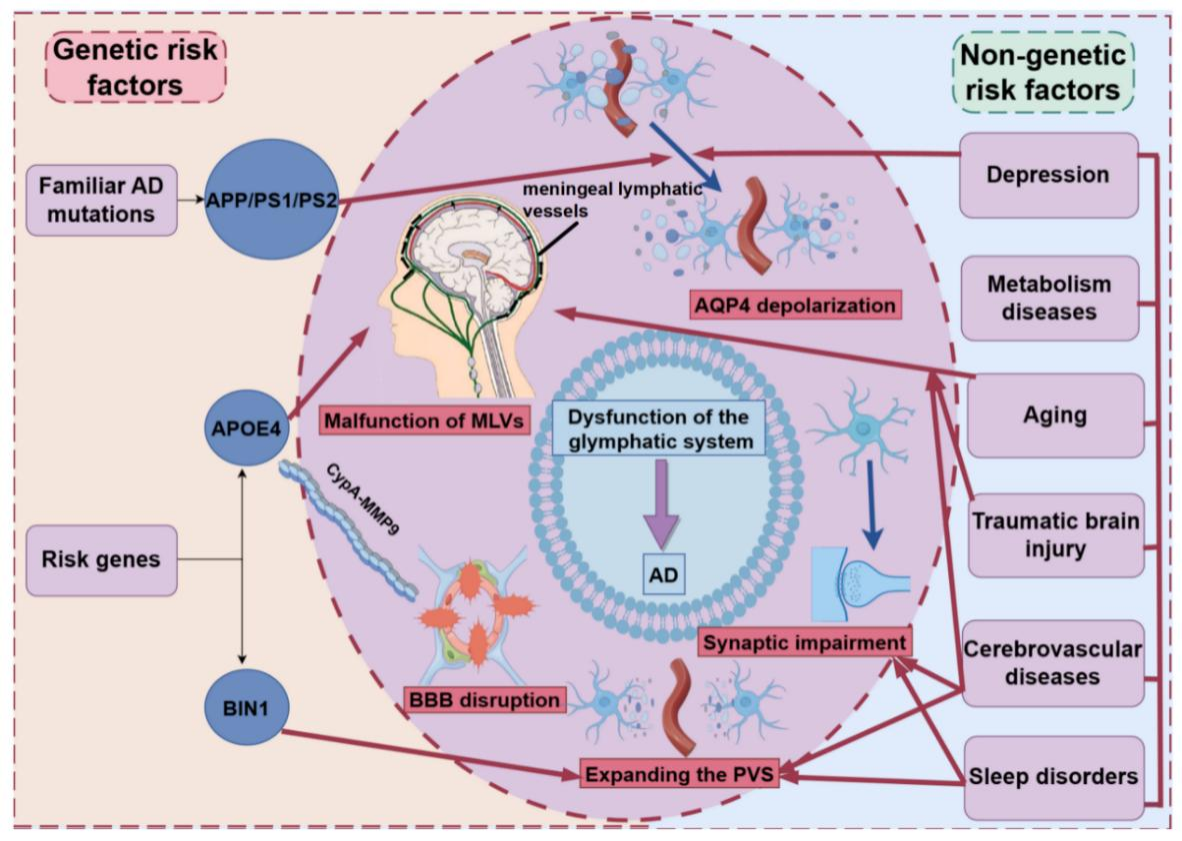
**The role of genetic and non-genetic risk factors in the dysfunction of glymphatic system during the progression of AD**. The dysfunction of the glymphatic system during the progression of AD can result in both genetic and non-genetic risk factors. Genetic risk factors, such as mutations in *APP*, *PS1*, and *PS2*, as well as *APOE4* and *BIN1*, can promote BBB disruption via CypA-MMP9, malfunction of MLVs, expansion of the PVS and depolarization of AQP4, all of which can lead to dysfunction of the glymphatic system. On the other hand, the expansion of PVS, malfunction of MLVs, synaptic impairment and depolarization of AQP4 are significantly affected by non-genetic risk factors, such as aging, sleep disorders, metabolic diseases, cerebrovascular diseases, traumatic brain injury and depression. Abbreviations: AD: Alzheimer’s disease; MLVs: meningeal lymphatic vessels; AQP4: aquaporin-4; APOE4: apolipoprotein E4; BBB: blood-brain barrier; PVS: paravascular space; APP: amyloid precursor protein; PS1: presenilin 1; PS2: presenilin 2; BIN1: bridging integrator 1; CypA-MMP9: CypA-nuclear factor-kB-matrix-metalloproteinase-9.

## 8. Conclusion and future directions

Genetic and non-genetic risk factors collectively contribute to dysfunction in the glymphatic system, thereby fostering the progression of AD through various mechanisms (Fig. 3). This review underscores how impairments in the glymphatic system can result from interference in the glymphatic pathway, obstruction of MLVs, disruption of the BBB and impairment of astrocyte synaptic function. All of these factors hasten the progression of AD. Genetic risk factors, such as mutations in APP, PS1, and PS2, as well as the APOE4 allele and a single-nucleotide polymorphism in BIN1, can promote BBB disruption via CypA-MMP9, malfunction of MLVs, expansion of the PVS and depolarization of AQP4, all of which can lead to dysfunction of the glymphatic system. On the other hand, the expansion of PVS, malfunction of MLVs, synaptic impairment and depolarization of AQP4 are significantly affected by non-genetic risk factors, such as aging, sleep disorders, metabolic diseases, cerebrovascular diseases, TBI and depression. Given that neurodegenerative diseases are often associated with the accumulation of abnormal proteins in the brain, enhancing the function of the glymphatic system could facilitate the clearance of these proteins. On the basis of the findings presented in this review, we propose that various approaches, such as promoting the production of CSF, maintaining the openness of the PVS, enhancing the polar distribution of AQP4, increasing the intercellular space in brain tissue, improving fluid flow dynamics, ensuring the drainage of MLVs, promoting the integrity of the BBB, and enhancing the synaptic function of astrocytes, could improve the function of the glymphatic system. This improvement may facilitate the clearance of abnormal proteins in the brain by regulating the glymphatic system. Similarly, to support the development of new preventive and diagnostic tools and to identify potential therapeutic targets, much work is needed to understand how glymphatic function is affected at both the behavioral and genetic levels and how this function is altered in human disease. Consequently, this research may inspire researchers to explore the workings of the glymphatic system as a guiding principle to identify novel targets for mitigating the onset and progression of AD.
